# Predicting Dental Anxiety and Cooperative Behavior in Children Using Machine Learning: A Cross-Sectional Predictive Modeling Study

**DOI:** 10.3390/dj14030170

**Published:** 2026-03-16

**Authors:** Narmin M. Helal, Heba Sabbagh

**Affiliations:** Pediatric Dentistry Department, King Abdulaziz University, Jeddah 21589, Saudi Arabia; hsabbagh@kau.edu.sa

**Keywords:** machine learning, pediatric dentistry, dental anxiety, cooperative behavior, predictive modeling

## Abstract

**Background/Objectives**: Dental anxiety and uncooperative behavior present significant challenges in pediatric dentistry and may adversely affect treatment outcomes and oral health. The main goal of this study was to evaluate the predictive performance of machine learning models in classifying dental anxiety measured using the Abeer Children Dental Anxiety Scale (ACDAS), predicting uncooperative behavior, estimating continuous dental anxiety scores, and identifying key predictors among children aged 6–11 years attending pediatric dental clinics in Jeddah, Saudi Arabia. **Methods:** This is an analytical cross-sectional study conducted among 952 children to evaluate whether machine learning models could predict dental anxiety and cooperative behavior based on demographic, clinical, and behavioral variables. Twenty variables captured demographic, medical, and dental history, BMI, and anxiety/behavioral measures. Data preprocessing included removing sparse variables, imputing missing values, and encoding categorical and ordinal variables appropriately. Logistic Regression models were trained to classify dental anxiety and cooperative behavior. A Random Forest Regressor was used to predict continuous anxiety scores, and a Random Forest Classifier was used for feature importance analysis. Principal Component Analysis (PCA) and K-Means clustering were applied to explore behavioral subgroups. **Results:** This dataset shows the Logistic Regression model with 0.92 accuracy (ROC AUC 0.98) for predicting dental anxiety and 0.91 accuracy (ROC AUC 0.95) for cooperative behavior. The Random Forest Regressor predicted anxiety scores with R^2^ = 0.97. Feature importance revealed that sensory and cognitive responses were key predictors of anxiety and cooperation. Unsupervised clustering identified two behavioral profiles: one with lower and another with higher anxiety and cooperation. **Conclusions:** ML models demonstrated strong prediction of dental anxiety and cooperation in this pediatric sample. While promising for early detection and personalized management of anxious or uncooperative children, further validation is essential before clinical use.

## 1. Introduction

Dental anxiety (DA) is an adverse response to dental treatment, often rooted in childhood trauma or negative habits [1]. It is closely linked to fear and avoidance, leading to poorer oral health [2]. About one-third of children experience some level of dental fear, with prevalence between 6% and 20% in those aged 4 to 18 [3]. Child dental anxiety contributes to avoidance, and lower quality of life [4]. In some cases, it may persist into adulthood, potentially developing into dental phobia, which delays treatment and requires more invasive procedures, further increasing fear [5].

Assessing dental fear and behavioral responses in children typically relies on a combination of self-reports, observational ratings, and structured interviews [6]. Tools such as the Facial Image Scale (FIS), Abeer Children Dental Anxiety Scale (ACDAS), Modified Dental Anxiety Scale (MDAS), and drawing-based or projective measures have demonstrated clinical utility in evaluating children’s emotional responses to dental treatment. However, these instruments are primarily designed for cross-sectional assessment and typically evaluate anxiety in isolation from broader socio-demographic, psychosocial, and behavioral factors that may also influence cooperation and treatment behavior in pediatric dental settings [3,7]. With ongoing technological advancements, there is growing interest in integrating artificial intelligence (AI) to enhance these assessments, allowing for more comprehensive analyses that incorporate demographic and socioeconomic variables, such as concerns about treatment costs, that influence children’s dental anxiety [8].

Building on these limitations, machine learning (ML), a powerful AI branch, has become an innovative predictive tool in healthcare [9]. ML approaches are capable of modeling complex interactions across multiple clinical, demographic, and behavioral variables and have shown promise in supporting risk prediction and outcome stratification in healthcare settings [10,11]. Within dental research, emerging studies have applied ML techniques to oral health outcomes and psychological indicators; however, their use in predicting behavioral cooperation and dental anxiety in children remains limited [12].

Despite growing interest in ML applications in dentistry, most existing studies have examined anxiety, behavioral outcomes, or psychosocial factors as isolated constructs or have focused on descriptive associations rather than predictive modeling. There is still a lack of comprehensive ML frameworks that combine socio-demographic, medical, anthropometric, and behavioral variables to jointly predict dental anxiety and cooperative behavior in pediatric patients. Developing such models could help identify behavioral risks earlier and guide personalized management strategies in clinical practice.

Accordingly, this study applied ML methods to a large pediatric dental dataset to (1) predict dental anxiety, (2) predict uncooperative behavior, (3) estimate continuous anxiety scores, (4) identify key predictors associated with anxiety and cooperation, and (5) explore behavioral subgroups using unsupervised learning. The primary objective was to evaluate the predictive performance and feasibility of ML approaches for early identification of at-risk children in pediatric dental settings.

## 2. Materials and Methods

### 2.1. Study Design

This is an observational, analytical, cross-sectional study with a predictive machine-learning secondary data analysis. The study’s large dataset includes data collected from 952 pediatric patients aged 6–11 years across various hospitals in Jeddah, Saudi Arabia. Data collection took place from December 2022 to November 2023. The dataset contains 70 different features with a wide range of parameters, including demographic information, medical and dental history, Body Mass Index (BMI) values, dental anxiety levels (as measured by the Abeer Children Dental Anxiety Scale—ACDAS), dental visit behavioral ratings, and additional sensory and cognitive responses [13].

### 2.2. Ethical Approval

Ethical approval for this study was obtained from King Abdulaziz University Faculty of Dentistry and the National Guard at King Abdul-lah International Medical Research Center Research Ethics Committees (Ethical Approval Number: 1779/23, Approval date on 29 January 2023). The whole research was conducted in compliance with the STROBE guidelines to ensure rigor as well as transparency of conduct and reporting.

### 2.3. Inclusion and Exclusion Criteria

Participants were selected to ensure a homogeneous and relevant dataset. Children aged 6 to 11 years who had never undergone invasive dental treatment with local anesthesia were included. Excluded were children with behavioral difficulties as defined by [14], developmental delays, mental disabilities, uncontrollable medical conditions, or those requiring urgent dental care during the study.

### 2.4. Data Preprocessing

Before developing the model, data were preprocessed to ensure quality, consistency, and suitability for machine learning. This involved removing irrelevant or highly sparse columns, imputing missing values with medians for numerical data and modes for categorical data, and converting categorical variables such as “Yes”/”No” responses into numerical form (e.g., 1/0). Variables with more than 40% missing data were excluded from analysis (e.g., Previous_Treatment_Type 60.5%, Phone_N 51.7%). To prevent target leakage, all outcome-derived or post-treatment variables were excluded from the predictor feature sets prior to model training. For the retained variables, the proportion of missing values ranged from 0% to 22.2% and was assumed to be missing at random. Median imputation was applied to numerical variables and mode imputation to categorical variables to preserve sample size while minimizing distributional distortion. Additionally, ordered categorical variables like Income, Parental Education, and BMI categories were transformed into appropriate numerical representations for use in predictive models.

### 2.5. AI Model Development and Evaluation

After thorough data preprocessing, various machine learning models were developed and validated across five tasks. Default model hyperparameters were used for all models, as the objective of this study was exploratory performance benchmarking rather than optimization. Model choices were based on data type (binary, multiclass, continuous) and their performance in healthcare predictions. Categorical predictors were one-hot encoded, and continuous features were standardized using StandardScaler prior to training logistic regression models to improve numerical stability and convergence. All analyses were performed in Python (version 3.13.0), utilizing the libraries scikit-learn, pandas, NumPy, and Matplotlib. Models and preprocessing artifacts were serialized using pickle and joblib to support reproducibility and future deployment.

#### 2.5.1. AI Model Evaluation

Classification models were evaluated using accuracy, precision, recall, F1-score, ROC–AUC, and confusion matrices. Regression performance was assessed using Mean Absolute Error (MAE), Root Mean Squared Error (RMSE), and R^2^. Outcome class distributions were examined prior to modeling. Because class imbalance was moderate and preserved through stratified sampling, no oversampling or class-weight adjustments were applied. This choice avoided the risk of distributional distortion and overfitting in a relatively balanced dataset.

#### 2.5.2. Dental Anxiety Classification (Binary)

To classify dental anxiety as a binary variable (Anxiety_Label), Logistic Regression was used because of its interpretability and robustness in modeling probabilities. Data was split into training (80%) and testing (20%) sets using stratified sampling to preserve the proportional distribution of anxiety and behavior outcome classes. Model performance was evaluated using accuracy, classification report (precision, recall, F1-score), confusion matrix, and ROC-AUC with corresponding ROC curves.

#### 2.5.3. Uncooperative Behavior Prediction (Binary)

Logistic Regression models were used to predict uncooperative behavior (Behavior_Label). Using the same preprocessing, data splitting, and evaluation methods as for dental anxiety classification ensured a consistent and robust assessment of the model’s ability to predict this behavior.

#### 2.5.4. Dental Anxiety Score Prediction (Continuous Regression)

A Random Forest Regressor was used to predict continuous Anxiety_Score due to its robustness, ability to model non-linear relationships, and resistance to overfitting, making it suitable for complex regression tasks. Model accuracy was evaluated using Mean Absolute Error (MAE), Root Mean Squared Error (RMSE), and R^2^ Score, which measure prediction error, emphasize larger errors, and indicate the variance explained, respectively.

#### 2.5.5. Feature Importance Analysis

A Random Forest Classifier was used to identify the key predictors of dental anxiety and non-cooperation. Its built-in method assessed each feature’s contribution to reducing impurity, such as Gini impurity. This analysis helps uncover the causes of these outcomes, offers clinical insights, and guides future research. The results, shown as bar plots, highlight the importance of different features in predicting outcomes and pinpoint key behavioral and environmental indicators. These importance values reflect model-specific impurity reduction and may be biased toward variables with higher cardinality; therefore, they should not be interpreted as causal factors, but rather as indicators of relative predictive contribution within the trained model.

#### 2.5.6. Unsupervised Learning: Behavioral Cluster Segmentation

Unsupervised learning used K-Means clustering to segment patient profiles based on their inherent characteristics. The optimal number of clusters (k) was determined via the Elbow Method, which plots the Within-Cluster Sum of Squares (WCSS) to identify the point of diminishing returns. Internal cluster validity was additionally examined using the silhouette coefficient to assess cohesion and separation between clusters. Subsequently, Principal Component Analysis (PCA) was applied only for two-dimensional visualization of cluster structure; clustering itself was performed on the full standardized feature set.

## 3. Results

### 3.1. Dataset Preprocessing

This study used data from 952 pediatric patients with 70 features, including demographics, medical history, dental history, BMI, and various anxiety and behavior metrics. The original dataset included 6 numerical, 4 integer, and 60 categorical features. Data preparation involved removing columns with over 40% missing data and irrelevant fields like Unnamed: 0, ID, and Phone_N. Missing values were filled using the mode for categorical and the median for numerical data. The data was then standardized by encoding ordered variables like Parental Education and Income numerically, and converting categorical values to a common format, such as “yes”/”no” to 1/0.

### 3.2. Key Features

The models used a comprehensive set of features to predict dental outcomes, categorized into key groups. Demographic features included age, gender, and socio-economic indicators. Medical and dental history covered past health conditions and experiences. Anxiety and behavior metrics, such as Anxiety_Score, Anxiety_Label, and Behavior_Label, were crucial. Other behavioral and cognitive features included responses to smells, feelings of losing control, perceptions of sounds, and general dispositions like shyness at the dentist. These features helped identify the nuanced drivers of anxiety and uncooperative behavior.

### 3.3. Initial Data Insights

Our initial visual analysis uncovered clear trends that informed our predictive model. Exploratory visual trends suggested a modest association between BMI and anxiety measures; however, BMI was treated only as a contextual variable and not as a predictive outcome. The overweight group had a higher median anxiety score and a broader score range. The results of this study revealed modest correlations between anxiety and behavioral indicators, which were used descriptively to contextualize the model interpretation. Additionally, there was a strong link between anxiety and behavioral metrics: a child’s anxiety score highly correlated with their anxiety label (r ≈ 0.76) and behavior label (r ≈ 0.64). This suggests a strong psychological connection between anxiety and behavioral reactions in dentistry. Although most children were categorized as “not scared,” females were somewhat more likely than males to respond as “scared,” as shown by a count plot of behavior by gender. This indicates possible gender differences in coping mechanisms. 

### 3.4. Machine Learning Model Performance

#### 3.4.1. Dental Anxiety Classification

**Figure 1:**
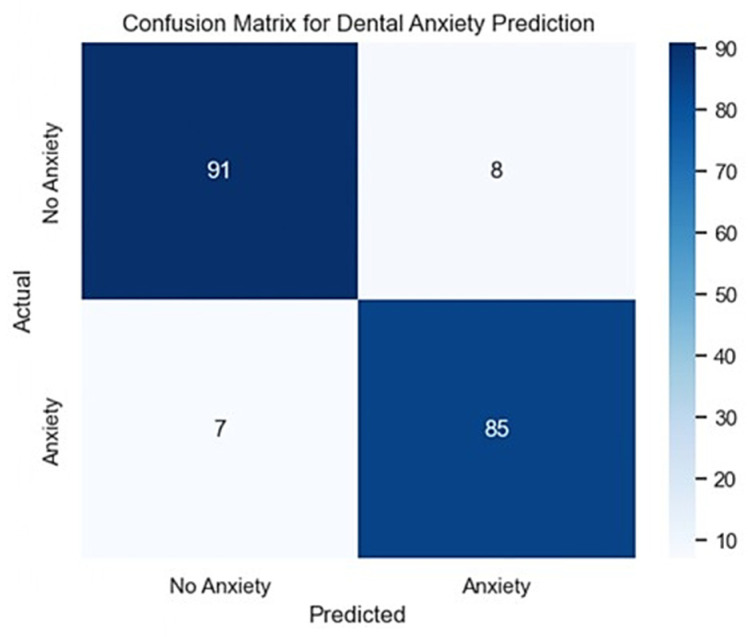
Confusion matrix illustrating the performance of the dental anxiety classification model across anxiety categories.

On the test set, this Logistic Regression model, which was developed to forecast dental anxiety (Anxiety_Label), had a high accuracy of 92%. In the hold-out test set, 99 children were classified as non-anxious (class 0) and 92 as anxious (class 1), for a total of 191 test cases. The model was able to classify the majority of patients, as shown in Figure 1. The impact and efficacy of the model are proven by its balanced precision, recall, and F1-score of 0.92 for the anxious and non-anxious classes. The model achieved an ROC–AUC of 0.97 on the hold-out test set, indicating excellent discrimination between anxious and non-anxious children. Thus, it guarantees that neither group is frequently mistakenly classified. The model demonstrated balanced classification performance on the hold-out test set; however, prospective clinical validation is required before implementation.

#### 3.4.2. Uncooperative Behavior Prediction

**Figure 2:**
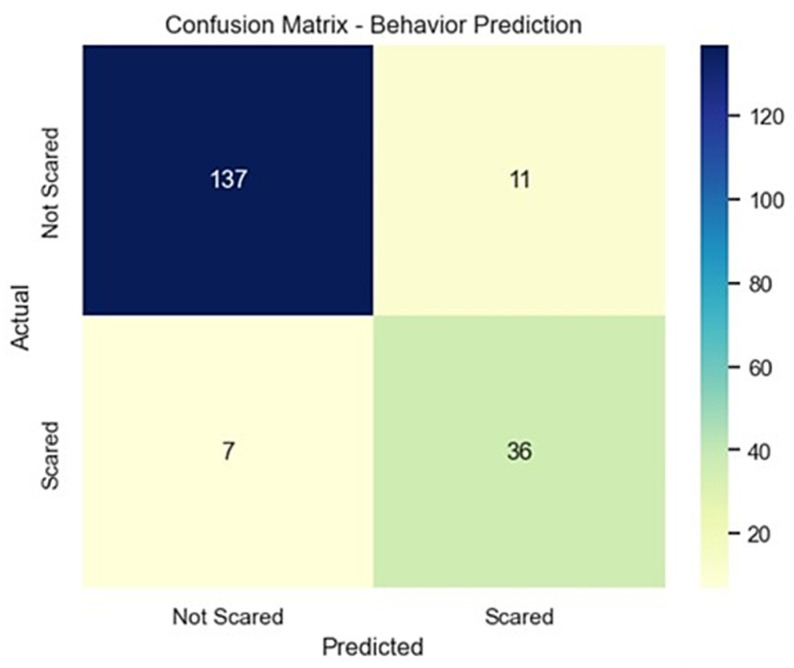
Confusion matrix displaying observed and predicted categories for the behavioral outcome model.

A second Logistic Regression model predicted uncooperative behavior (Behavior_Label) with 91% accuracy. The test set contained 99 cooperative children (class 0) and 92 uncooperative children (class 1), reflecting the same stratified class distribution as in the anxiety model. It was highly effective at identifying cooperative children (precision: 0.95, recall: 0.93). The confusion matrix in Figure 2 shows its performance. It was slightly less accurate for scared children (recall: 0.84), likely due to fewer in the dataset. The model achieved a ROC–AUC of 0.96 on the hold-out test set, demonstrating strong capacity to distinguish cooperative from uncooperative children. The model is reliable for testing cooperation but may require calibration to better identify at-risk, uncooperative children.

#### 3.4.3. Dental Anxiety Score Prediction

**Figure 3:**
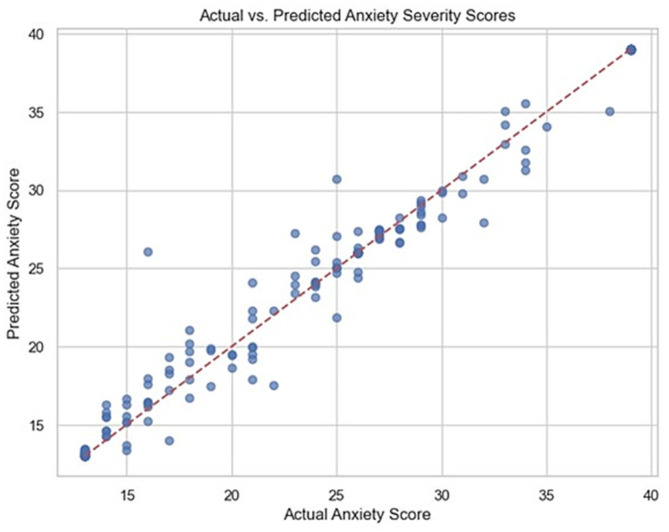
Scatter plot comparing actual and predicted anxiety severity scores with reference alignment along the identity line.

A Random Forest Regressor accurately predicted a child’s anxiety severity, achieving an R^2^ of 0.97, which indicates it explained 97% of the variance. Despite the high R^2^ value, the model was evaluated only on the hold-out test set and not on an external dataset; therefore, the results should be interpreted cautiously. Low MAE (0.74) and RMSE (1.95) confirmed precise predictions. Error metrics are reported in the original anxiety score units. The scatter plot in Figure 3 supported the model’s high accuracy, enabling clinicians to quantify anxiety more precisely and personalize care.

#### 3.4.4. Feature Analysis

A Random Forest Classifier identified key factors influencing anxiety and behavior. For dental anxiety, sensory and cognitive responses such as reactions to smells, feelings of losing control, and responses to instrument sounds, were most influential. Similarly, predictors of uncooperative behavior included shyness, expected behavior, and losing control. By focusing on these triggers, clinicians can develop a data-driven approach. For example, pediatric dentists can prepare children for specific sensory experiences to proactively reduce anxiety.

#### 3.4.5. Behavioral Cluster Segmentation

**Figure 4:**
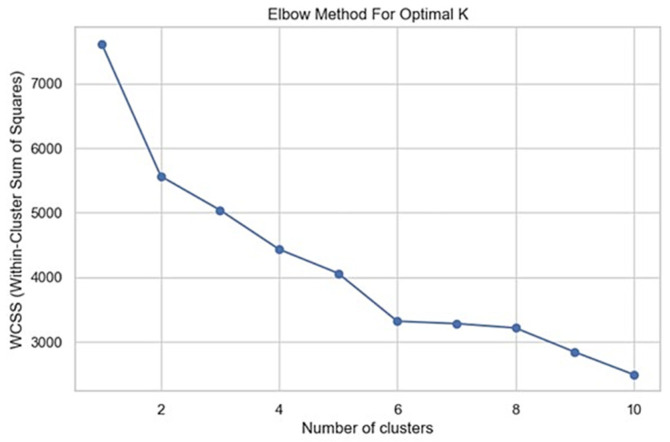
Elbow method plot showing the change in within-cluster variance across values of k to identify the optimal cluster number.

Our study identified two behavioral groups among children using an unsupervised K-Means model. The Elbow Method indicated that two clusters best fit the data, enabling effective patient stratification without predefined labels. Cluster structure was interpreted descriptively, as this is an exploratory analysis. The first group, “Confident Responders,” consisted of children less afraid of dental stimuli and more cooperative, as shown in Figure 4.

**Figure 5:**
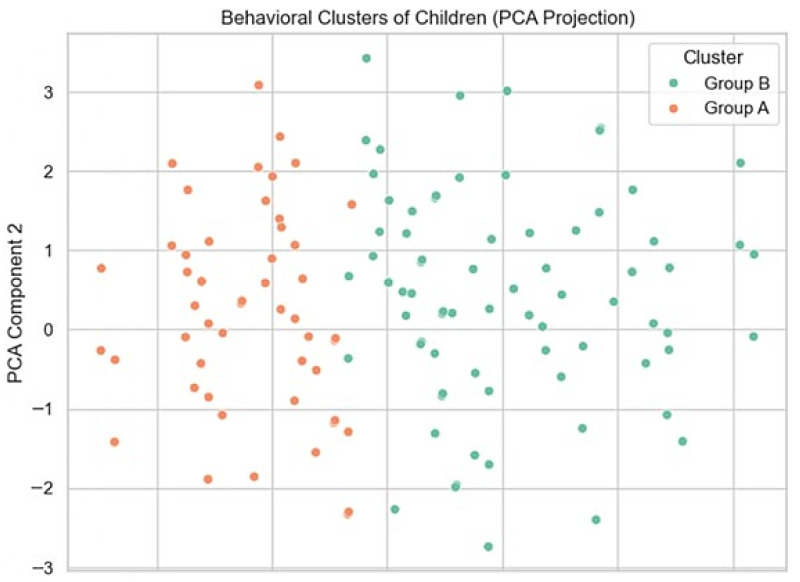
PCA projection showing the distribution and separation of behavioral clusters in two-dimensional space.

The second, “Sensitive Responders,” included children with lower cooperation, increased shyness, and higher anxiety. As shown in Figure 5, this segmentation can improve patient management by allowing clinicians to adapt communication and treatment strategies to each child’s behavioral profile. For instance, less preparation may suffice for “Confident Responders,” while “Sensitive Responders” may benefit from more pre-treatment guidance.

## 4. Discussion

The findings of this study suggest that machine learning approaches may have potential utility in pediatric dental care and contribute to the growing literature on predictive modeling of dental anxiety and uncooperative behavior in children. Our results further support the potential utility of AI-based approaches in this domain but also offer clinically relevant insights by identifying key predictive features and distinguishing distinct behavioral profiles. The Logistic Regression models’ high accuracy (0.92 for dental anxiety and 0.91 for uncooperative behavior) and impressive ROC AUC scores (0.97 and 0.96, respectively) demonstrated high classification performance of children at risk. These results align with previous studies demonstrating the utility of machine learning in predicting healthcare outcomes, such as those by Norouzi and Machado (2024) [15], who achieved high accuracy in predicting general anxiety, and Nemesure et al. (2021) [16], who employed a stacked ensemble classifier for GAD prediction.

Although direct comparisons are difficult due to varying contexts and datasets, our findings are particularly relevant to the specific challenges associated with pediatric dental anxiety and behavior. The ability of a relatively simple, interpretable model like Logistic Regression to achieve such high performance suggests a clear linear or near-linear relationship between the identified features and the binary outcomes, providing practical utility for clinicians. While the performance of Random Forest Regressor on the prediction of continuous anxiety scores with R^2^ 0.971 reflects strong predictive performance in quantifying the level of anxiety. This high predictive performance should be interpreted cautiously, as behavioral and psychological outcomes are inherently complex and external validation was not performed, suggesting that the selected feature set contains clinically meaningful information relevant to anxiety prediction; however, as the models were not externally validated, these results should be interpreted cautiously.

In this study, BMI was included as an anthropometric covariate to contextualize anxiety and behavioral responses, rather than being modeled as a predictive outcome. Exploratory trends were consistent with prior research suggesting a potential association between BMI and dental anxiety; however, no causal interpretation is implied [13]. The role of BMI in pediatric dental behavior should therefore be viewed as hypothesis-generating and warrants further investigation in larger, longitudinal and multicenter samples. The robust performance of Random Forest models across classification and regression tasks aligns with their reputation for high precision and stability with structured, heterogeneous data, as noted by Mohajeri et al. (2024) [17]. The feature importance analysis provided critical insights into the underlying drivers of anxiety and uncooperative behavior. Identifying factors like a child’s reaction to instruments and previous treatment experiences as prominent predictors supports existing evidence that these experiences may be associated with initial dental encounters. This echoes the proofs from the literature review regarding past painful experiences strongly correlating with future dental anxiety [5]. Thus, those insights can guide targeted interventions, allowing clinicians to focus on specific triggers that exacerbate a child’s apprehension.

Furthermore, the unsupervised learning component, which clustered children into “Confident Responders” and “Sensitive Responders,” is a novel direction for personalized care. The clustering approach is a data-driven method to categorize patients beyond conventional scales, allowing for the development of personalized behavioral management strategies. This aligns with the overall application of machine learning in healthcare to foster precision medicine and treatment protocols [18]. Thus, this work significantly enhances dental anxiety and behavior management and comprehension among children through the implementation of a comprehensive machine learning approach. Unlike previous studies that often disentangle correlations [13] or aim at specific factors like dentist fear through drawing analysis [3], our models make simultaneous predictions for several outcomes based on a broad set of demographic, medical, and behavioral parameters.

The methodology, from rigorous data preprocessing to the selection of appropriate ML algorithms, demonstrates that machine learning can model potentially complex and non-linear relationships that may be less easily captured using conventional statistical approaches. Despite their significant contributions, some limitations must be acknowledged. The cross-sectional design effectively identifies associations but cannot establish causation. The dataset, sourced from Jeddah, Saudi Arabia, and limited to children aged 6–11, restricts the applicability of the models to other populations and age groups. Although comprehensive, relying on a single source means the models have not been externally validated. Subjective assessment tools, such as observation scales requiring skilled examiners, may introduce ambiguity into the data. Future research should focus on longitudinal studies to evaluate the models’ long-term predictive stability and impact. External validation across diverse populations and regions is essential for broader generalizability. Exploring real-time clinical implementation of these models offers promising benefits for patient care. Incorporating advanced data analysis, such as deep learning of behavioral or physiological traits (e.g., drawing analysis; [3]), could further enhance predictive accuracy and personalized treatment. To address these limitations, future work should include prospective, longitudinal validation of the models, expanding datasets geographically and demographically, and developing a user-friendly clinical application. Such a tool would enable dental professionals to input patient data and receive immediate, evidence-based predictions for anxiety, behavior, and BMI, facilitating personalized, proactive care.

## 5. Conclusions

This study suggests the potential utility of AI in pediatric dentistry, demonstrating high predictive performance of machine learning models for dental anxiety and uncooperative behavior in children aged 6–11 years in Jeddah, Saudi Arabia. Utilizing a comprehensive set of socio-demographic, medical, anthropometric, and behavioral predictors, the models identified key features influencing these behaviors and classified children into behavioral profiles through unsupervised learning. These findings suggest ML approaches could support early identification of at-risk pediatric patients. However, due to the cross-sectional design and lack of external validation, further prospective and multicenter studies are needed to confirm clinical applicability.

## Data Availability

The data presented in this study are available on request from the corresponding author.

## Abbreviations

The following abbreviations are used in this manuscript:

DA	Dental Anxiety
ACDAS	Abeer Children Dental Anxiety Scale
FIS	Facial Image Scale
MDAS	Modified Dental Anxiety Scale
AI	Artificial Intelligence
ML	Machine Learning
PCA	Principal Component Analysis
ROC AUC	Receiver Operating Characteristic—Area Under the Curve
R^2^	Coefficient of Determination
MAE	Mean Absolute Error
RMSE	Root Mean Squared Error
WCSS	Within-Cluster Sum of Squares
STROBE	Strengthening the Reporting of Observational Studies in Epidemiology
DSR	Deanship of Scientific Research (at King Abdulaziz University)
